# Supplementary material for: Factors Associated with Decisions for Initial Dosing, Up-Titration of Propiverine and Treatment Outcomes in Overactive Bladder Syndrome Patients in a Non-Interventional Setting
Source: J Clin Med. 2021 Jan 15;10(2):311. doi: 10.3390/jcm10020311 (PMC7830207; doi:10.3390/jcm10020311)

Online Supplement to

Factors associated with decisions for initial dosing, for up-titration of propiverine and treatment outcomes in overactive bladder syndrome patients in a non-interventional setting

Marjan Amiri, Tim Schneider, Matthias Oelke, Sandra Murgas, Martin C. Michel

This Supplement provides three types of data:

- additional data related to both studies
- data for study II where analogous data for study I are presented in main manuscript
- data related to the primary aim of study II (exploration of role of additional material).

The section numbering follows that of the main manuscript.

2. Methods

Study II was primarily designed to explore the effects of additional material (information sheet about OAB and mode of action of the drug) on efficacy/tolerability and premature discontinuation rate in a treatment period of 12 weeks; in contrast to study I, it allowed extension of observation for up to 24 weeks in a subgroup of patient. Thus, both groups were informed verbally about their condition, bladder training and therapy at visit 1 according to standard information provided by a given physician; the second group additionally received written information (leaflet) on bladder training and therapy. Allocation to one of the two groups was made by the physician in a consecutive manner. Based upon a planned number of 1000 participants, it was intended that half of them were followed for 24 weeks based on physician's judgment. When it became obvious that recruitment targets could not be reached, the study was ended after a total recruitment of 745 patients including 363 with a 24 weeks observation period. Other aspects of the study design were very similar to those of study I as reported in the main manuscript unless stated explicitly below; therefore, data from both studies were used for the scientific questions defined in the main manuscript.

3. Results

3.1. Patient flow and baseline data

Supplementary Figures 1 and 2 show patient flow in studies I and II, respectively.

Supplementary Table 1 shows demographics and baseline OAB-related variables of the overall population of studies I and II.

Many patients in studies I and II had undergone previous documented interventions with implications for lower urinary tract function, including hysterectomy (n = 260 and 99, respectively), transurethral prostatectomy (123 and 53), radical prostatectomy (57 and 28), cystopexy (46 and 32), incontinence surgery (26 and 14), transurethral bladder resection (10 and 8), Caesarean section (5 and 0), brachytherapy (5 and 1) or radiotherapy of the prostate (5 and 1).

The most reported comorbidities in studies I and II were typical for the age group and included as most frequently reported hypertension (n = 675 and 429, respectively), diabetes mellitus (291 and 189) and coronary heart disease (150 and 115); a coexisting benign prostate

enlargement was documented for 128 and 83 men, respectively. Accordingly, many patients also reported comedications including as most frequently used drugs inhibitors of the renin-angiotensin system (238 and 170), β-adrenoceptor antagonists (236 and 121) and antidiabetics (232 and 156). Many patients reported previous OAB treatment (35.7% and 39.2%).

3.2. Descriptive analysis of treatment outcomes

Supplementary Figure 3 shows treatment outcomes in the 30/30, 30/45 and 45/45 group of study I depicted as means ± SD to enable easier comparison with previously published studies.

While no specific instructions on time of administration were given, most patients in study II 443/624 (71%) reported taking propiverine in the morning. After 4 weeks of treatment, clinically meaningful improvements (median with IQR; followed by mean ± SD) were observed in the overall efficacy population with a reduction in urgency episodes by 4 (2; 7 ; 4.7 ± 4.1), in incontinence episodes by 2 (1; 4; 2.8± 3.2) in micturitions by 3 (2; 5; 3.8± 3.0) , and in nocturia episodes by 1 (1; 2; 1.4 ± 1.2). Between weeks 4 and 12, the overall cohort of patients reported additional improvements in urgency episodes by 1 (0; 3; 1.8±2.5), in incontinence episodes by 1 (0; 2; 1.0±1.6), in frequency by 1 (0; 3; 1.5±1.9), and in nocturnal voids by 0 (0; 1 ; 0.5± 0.9). Thus, overall improvements from baseline to week 12 were improvements of urgency episodes by 3 (1; 5; 3.5±3.1), in incontinence episodes by 1 (0; 3; 1.7±2.1), in frequency by 8 (6; 10; 8.2± 2.9) and in nocturnal voids by 1(1; 2 ; 1.7±1.2). Supplementary Figures 4 and 5 show treatment outcomes in the 30/30, 30/45 and 45/45 group of study II depicted as medians with IQR and as means ± SD, respectively.

The primary aim of study II had been to explore whether providing additional information material at visit 1 affects treatment outcomes at visit 3. Patient without and with the additional information material had very similar baseline values for their OAB symptoms: urgency: 10 (6; 8; 10.1 ± 5.3) vs. 9 (6; 12; 9.4 ± 5.9); incontinence: 3 (0; 6; 4.0 ± 4.3) vs. 3 (0; 6; 3.8 ± 3.9); frequency: 13 (10; 16; 13.3 ± 4.3) vs. 13 (10; 15; 13.0 ± 4.2); nocturia: 3 (2; 4; 3.5 ± 1.8) vs. 3 (2; 4; 3.3 ± 1.8). They also had very similar improvements relative to those baselines: urgency (without ; 6 (-9; -3; -6.6 ± 4.6) vs. -6 (-8; -3; -6.3 ± 5.4), incontinence -2 (-4; 0; -2.6 ± 3.5) vs. -2 (-4; 0; -2.6 ± 2.9); frequency: -5 (-7; -3; -5.2 ± 3.7) vs. -6 (-8; -3; -6.3 ± 5.4); nocturia: -2 (-2; -1; -1.8 ± 1.6) vs. -1 (-2; -1; -1.7 ± 1.4). Premature discontinuation, i.e. stopping treatment before visit 3, occurred in 24/385 (6.2%) and in 29/360 (8.1%).

3.3. Factors associated with Initial dosing decision at visit 1 (study II)

Demographic and OAB-related baseline data of subgroups receiving different initial doses are summarized in Supplemental Table 2. The two groups had similar mean age and height, but those starting on 45 mg had greater mean body weight (+5.2 kg) and BMI (+1.1 kg/m^2). The 45 mg group also had greater baseline symptom intensity for frequency and nocturia, without major differences in urgency and incontinence.

3.4. Factors associated with dosing increase at visit 2 (study II)

86% of patients having started with a dose of 30 mg remained on that dose (30/30 group), whereas 12% were switched to the 45-mg dose (30/45 group). 93% of patients having started with a dose of 45 mg remained on that dose (45/45 group), and 5% reduced it to 30 mg; the

latter group was not considered further as it was deemed too small to allow meaningful analysis.

Supplementary Table 3 shows demographic and OAB-related data at baseline and after 4 weeks of treatment in the 30/30 and the 30/45 group of study II. Patients with dose-escalation were slightly taller (+2.1 cm) and heavier (+3.8 kg) and had a greater BMI (+0.6 kg/m^2). They had a longer duration of OAB, and more daily urgency, micturition and nocturia episodes. These differences were maintained after 4 weeks of treatment.

3.5. Factor associated with treatment outcomes (study II)

Demographics and baseline values for the 30/30 and 30/45 groups in study II are shown in Supplementary Table 3 and those for the 45/45 group in Supplementary Table 2. Values of OAB parameters after 4 weeks of treatment are shown in Supplementary Table 3 for the 30/30 and the 30/45 group; for the 45/45 group, they were 4.5 (5.1 ± 3.7) for urgency, 2 (2.6 ± 0.6) for incontinence, 10 (10.2 ± 3.4) for frequency and 2 (2.4 ± 1.4) for nocturia

4. Discussion specific to study II

The primary aim of study II had been to explore the effect of additional written information provided at visit 1 on treatment outcomes. Our data do not support the idea that providing additional written information material improves treatment outcomes or reduces premature discontinuation rates to a clinically meaningful extent. Two previous studies have also looked at related questions. One of them was a randomized open-label study to explore as a secondary aim whether additional provision of a packet of educational materials enhances the health-related quality of life of OAB patients receiving transdermal oxybutynin (Sand et al., 2007). This study ad used a package of information material including an educational booklet, OAB newsletters, dosing reminders, calendar reminder stickers and a bladder diary. The other was also a randomized study with a primary aim of testing whether a structured tool for self-education of OAB patients (Self-Assessment of Goal Achievement; SAGA) affected outcomes of treatment with fesoterodine (Schneider et al., 2014); due to differences in recruitment, this study did not reach the number of patients that the power calculations had suggested and remained inconclusive. Despite these differences, these two and the present study all concluded that provision of additional information material did not affect treatment outcomes with a muscarinic receptor antagonist in a clinically meaningful way.

5. References

Sand P, Zinner N, Newman D, Lucente V, Dnochowski R, Kelleher C, Dahl NV (2007) Oxybutynin transdermal system improves the quality of life in adults with overactive bladder: a multicentre, community-based, randomized study. BJU Int 99: 836-844. DOI 10.1111/j.1464-410X.2006.06658.x

Schneider T, Arumi D, Crook TJ, Sun F, Michel MC (2014) An observational study of patient satisfaction with fesoterodine in the treatment of overactive bladder: effects of additional educational material. Int J Clin Pract 68: 1074-1080. DOI 10.1111/ijcp.12450

6. Supplementary tables

Table 1: Demographic and OAB-related baseline variables in studies I and II. Data are shown as % of patients for gender (does not add up to 100 due to missing values) and as means ± SD for quantitative demographic parameters, and medians with inter-quartile ranges of OAB-related parameters (means ± SD also shown to facilitate comparison with previously reported studies). Note that 398 and 493 participants (29.8% and 66.2%) in studies I and II reported 0 incontinence episodes, i.e. were dry; the episode frequency of incontinence is based only on patients reporting at least 1 incontinence episode/24 h at baseline.

	**Study I**	**Study II**
n	1335	745
**Demographic parameters**		
Gender, % male/female	32.7/64.2	36/60
Age, years	65.7 ± 13.0	66.1 ± 13.7
Height, cm	169.2 ± 8.0	169.7 ± 7.8
Weight, kg	77.9 ±14.8	78.7 ± 14.6
BMI, kg/m^2	27.2 ± 4.6	27.3 ± 4.5
**OAB-related parameters**		
OAB duration, months	12.1 (4.0; 34.6)	17.2 (5.9; 47.9)
Urgency episodes/24 h	9 (6; 13; 10.1 ± 5.8)	9 (6; 12; 9.6 ± 5.4)
Incontinence episodes/24 h	4 (2; 6; 5.0 ± 3.9)	4 (2; 7; 5.2 ± 3.8)
Urinary frequency/24 h	13 (11; 16; 13.5 ± 4.2)	13 (10; 15; 13.1 ± 4.2)
Nocturia episodes/24 h	3 (2; 4; 3.4 ± 1.6)	3 (2; 4; 3.5±1.7)

Supplementary Table 2: Demographic and OAB-related baseline variables in patients starting treatment with a propiverine dose of 30 or 45 mg/d in study II. Data are shown as % of patients for gender (does not add up to 100 due to missing values) and as means ± SD for quantitative demographic parameters, and medians with IQR of OAB-related parameters (means ± SD only shown to facilitate comparison with previous reports). Descriptive p-values for the difference between groups are from univariate analysis using unpaired, two-tailed Kruskal-Wallis tests. The analysis of the OAB-symptoms included only patients that had a documented dose and a measured value at baseline other than 0.

	**Initial 30 mg**	**Initial 45 mg**	**p-value**
n	531	200	
**Demographic parameters**			
Gender, % female/male	60.6/34.5	57.0/38.5	
Age, years	66.4 ± 13.7	65.3 ± 13.8	0.4127
Height, cm	169.4 ± 7.7	170.4 ± 8.2	0.1145
Weight, kg	77.5 ± 13.7	82.3 ± 16.5	0.0004
BMI, kg/m^2	27.1 ± 4.3	28.2 ± 4.8	0.0007
**OAB-related parameters**			
Urgency episodes, per day	9 (6; 12)<br>9.5 ± 5.6	10 (6; 13)<br>10.1 ± 5.3	0.1183
Incontinence episodes, per day	4 (2; 7)<br>5.1 ± 3.8	4 (3; 7)<br>5.4 ± 3.8	0.2318
Urinary frequency, per day	12 (10; 15)<br>12.7 ± 4.4	14 (12; 16)<br>14.2 ± 4.0	<0.0001
Voiding per night (nocturia)	3 (2; 4)<br>3.3 ± 1.7	4 (3; 5)<br>3.9 ± 1.6	<0.0001

Supplementary Table 3: Demographic and OAB-related variables at baseline and after 4 weeks in patients starting treatment with a propiverine dose of 30 mg/d and either staying on that dose after 4 weeks or increasing it 45 mg/d. Data are shown as % of patients for gender, as means ± SD for continuous demographic parameters, and medians with IQR of OAB-related parameters (means ± SD only shown to facilitate comparison with previous reports). Descriptive p-values for the difference between groups are from univariate analysis using unpaired, two-tailed Kruskal-Wallis tests. The analysis of the OAB-symptoms included only patients that had a documented dose and a measured value at baseline other than 0.

	**Stay on 30 mg**	**Increase to 45 mg**	**p-value**
n	435	59	
**Demographic parameters**			
Gender, % female/male	64.0/36.0	60.7/39.3	
Previous OAB treatment, %	34.5	44.6	
Age, years	66.1 ± 12.9	65.1 ± 11.9	0.6651
Height, cm	169.2 ± 7.6	171.3 ± 8.0	0.0336
Weight, kg	77.2 ± 13.4	81.0 ± 15.3	0.0006
BMI, kg/m^2	27.0 ± 4.3	27.6 ± 4.7	0.0053
**OAB-related parameters at baseline**			
OAB duration, months	13.7 [5.5; 44.3]	22.6 [4.9; 55.9]	0.0107
Urgency episodes/24 h	8 [5; 12]<br>9.1 ± 5.0	10 [7; 14]<br>11.2 ± 7.3	0.0322
Incontinence episodes/24 h	4 [2; 7]<br>5.1 ± 3.9	4 [2; 6]<br>4.9 ± 3.7	0.7727
Urinary frequency/24 h	12 [10; 15]<br>12.5 ± 4.2	13 [12; 18]<br>14.6 ± 4.0	<0.0001
Nocturia/24 h	3 [2; 4]<br>3.3 ± 1.7	3 [3; 4]<br>3.6 ± 1.7	<0.0001
**OAB-related parameters after 4 weeks**			
Urgency episodes/24 h	4 [2; 6]<br>4.7 ± 3.6	8 [4; 10]<br>7.7 ± 4.9	
Incontinence episodes/24 h	2 [0; 4]<br>2.4 ± 2.5	3 [1; 5]<br>3.6 ± 3.8	
Urinary frequency/24 h	9 [7; 10]<br>8.9 ± 3.2	11 [9; 13]<br>11.6 ± 3.2	
Nocturia/24 h	2 [1; 2]<br>2.0 ± 1.3	3 [2; 3]<br>2.7 ± 1.3	

Supplementary Table 4: Factors associated with overall improvement of frequency (12 weeks vs. baseline) in a logistic regression analysis taking demographics, OAB parameters at baseline, duration of condition and dose level in to consideration. P-values for gender relate to male and those for dose level relate to the 45/45 group as reference; *: term not uniquely estimable.

Parameter	**Estimate ± SE**	**p-value**	**Estimate ± SE**	**p-value**
	**Study I**		**Study II**	
Gender, female	-0.200 ± 0.251	0.4259	-0.054 ± 0.370	0.8841
Age, years	0.029 ± 0.007	<0.0001	0.020 ± 0.011	0.0652
Weight, kg	-0.065 ± 0.064	0.3215	-0.049 ± 0.098	0.6235
Height, cm	0.071 ± 0.064	0.2729	0.050 ± 0.098	0.6104
BMI, kg/m^2	0.186 ± 0.187	0.3192	0.145 ± 0.293	0.6213
OAB duration, months	0.011 ± 0.002	<0.0001	0.002 ± 0.003	0.3542
Urgency/24 h	-0.015 ±0.020	0.4576	0.096 ± 0.030	0.0017
Incontinence/24 h	-0.042 ± 0.028	0.1312	0.081 ± 0.038	0.0367
Micturitions/24 h	-0.593 ± 0.019	<0.0001	-0.478 ± 0.048	<0.0001
Nocturia/24 h	-0.314 ± 0.069	<0.0001	-0.299 ± 0.102	0.0037
Dose 30/30*	-0.244 ± 0.236	0.3029	-0.273 ± 0.292	0.3498
Dose 30/45*	0.207 ± 0.297	0.4862	0.224 ± 0.437	0.6083

Supplementary Table 5: Factors associated with overall improvement of nocturia (12 weeks vs. baseline) in a logistic regression analysis taking demographics, OAB parameters at baseline, duration of condition and dose level in to consideration. P-values for gender relate to male and those for dose level relate to the 45/45 group as reference; *: term not uniquely estimable.

Parameter	**Estimate ± SE**	**p-value**	**Estimate ± SE**	**p-value**
	**Study I**		**Study II**	
Gender, female	-0.082 ± 0.094	0.3823	0.178 ± 0.143	0.2142
Age, years	0.012 ± 0.003	<0.0001	0.016 ± 0.004	0.003
Weight, kg	-0.011 ± 0.024	0.6558	-0.017 ± 0.039	0.6653
Height, cm	0.019 ± 0.024	0.4246	0.032 ± 0.038	0.4012
BMI, kg/m^2	0.033 ± 0.070	0.6318	0.053 ±0.113	0.6398
OAB duration, months	0.004 ± 0.001	<0.0001	0.001 ± 0.001	0.4056
Urgency/24 h	-0.008 ± 0.007	0.3039	-0.040 ± 0.112	0.0006
Incontinence/24 h	0.002 ± 0.010	0.8257	0.040 ± 0.015	0.0071
Micturitions/24 h	0.010 ± 0.011	0.3671	0.025 ± 0.019	0.1851
Nocturia/24 h	-0.765 ± 0.027	<0.0001	-0.657 ± 0.040	<0.001
Dose 30/30*	-0.098 ± 0.088	0.2666	-0.189 ± 0.113	0.0950
Dose 30/45*	0.033 ± 0.112	0.7685	-0.000 ± 0.170	0.9995

7. Legends to the supplementary figures

Supplementary Figure 1: Patient disposition in study I. Two patients starting on 30 mg were down-titrated to 15 mg at visit 2; 15 and 1 patients from the groups starting at 30 and 45 mg/d, respectively, had missing values on dosing after visit 2.

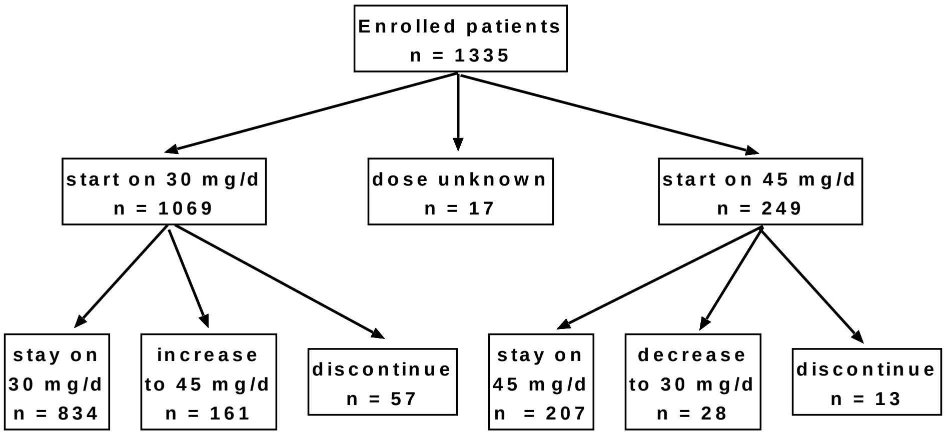

Supplementary Figure 2: Patient disposition in study II. 1 patient started at 15 mg. 15 patients from the group starting at 30 mg/d had missing values on dosing after visit 2.

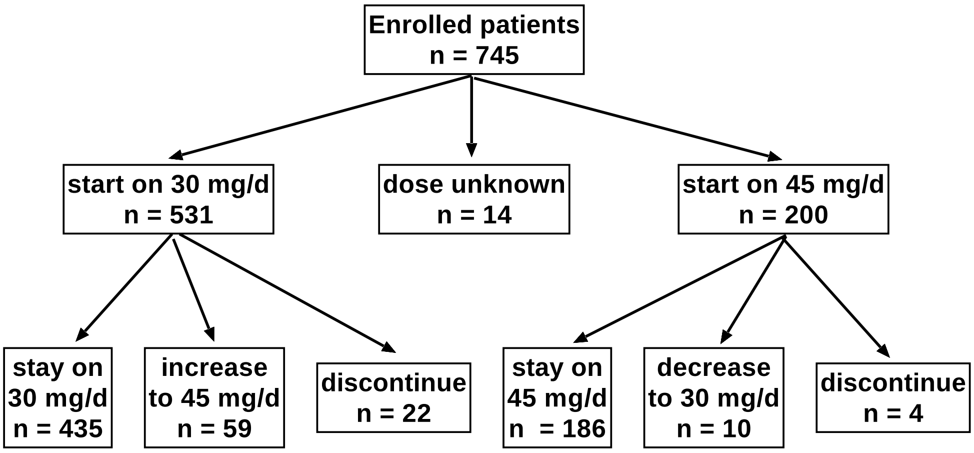

Supplementary Figure 3: Intra-individual change of OAB symptoms in the cohorts of patients starting on 30 mg and staying on that dose (30/30), starting on 30 mg and escalating to 45 mg at visit 2 after about 4 weeks (30/45) and starting and staying on 45 mg until study end after about 12 weeks (45/45) in study I. Patients not exhibiting a given symptom at baseline were excluded from the analysis of that symptom; specifically, 364 subjects reported no incontinence at baseline. Data are shown as means ± SD; medians wit IQR are shown in the main paper.

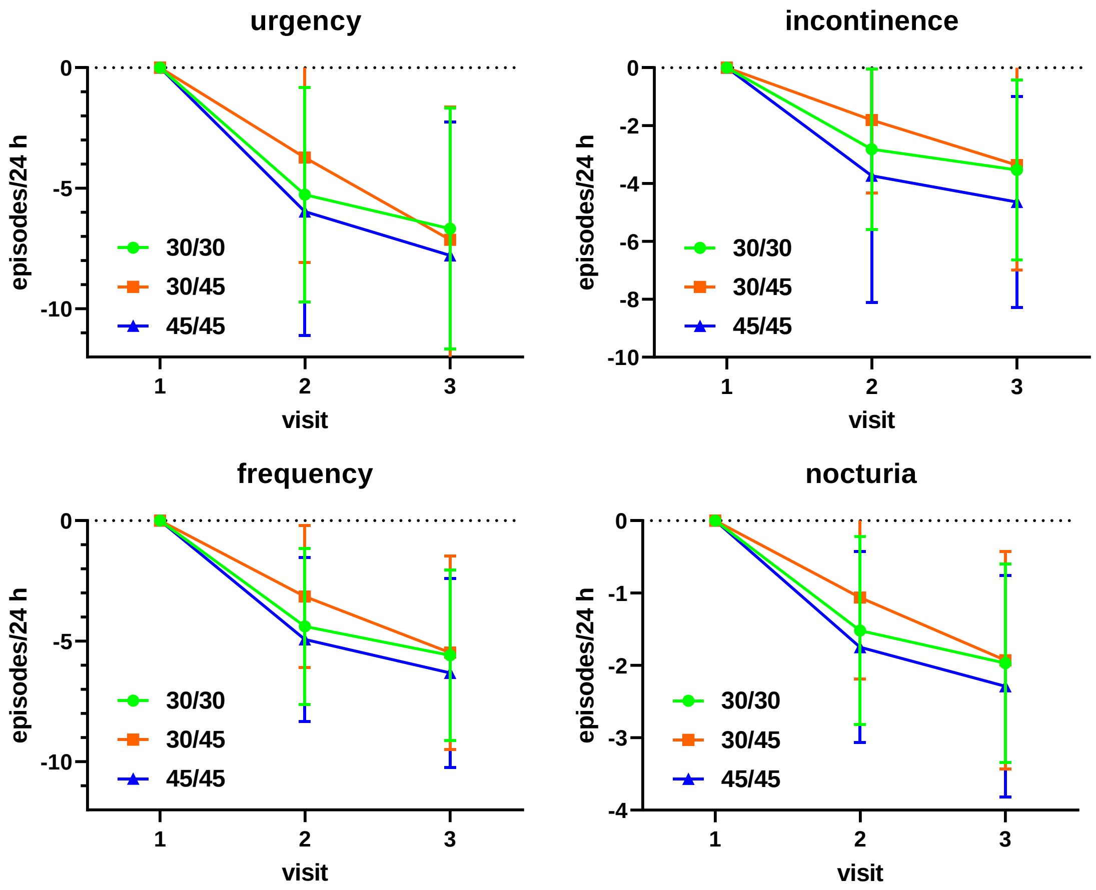

Supplementary Figure 4: Intra-individual change of OAB symptoms in the cohorts of patients starting on 30 mg and staying on that dose (30/30), starting on 30 mg and escalating to 45 mg at visit 2 after about 4 weeks (30/45) and starting and staying on 45 mg until study end after about 12 weeks (45/45) in study II. Patients not exhibiting a given symptom at baseline were excluded from the analysis of that symptom; specifically, 171 subjects reported no incontinence at baseline. Data are shown as medians with IQR; means ± SD are shown for comparison in Supplementary Figure 5.

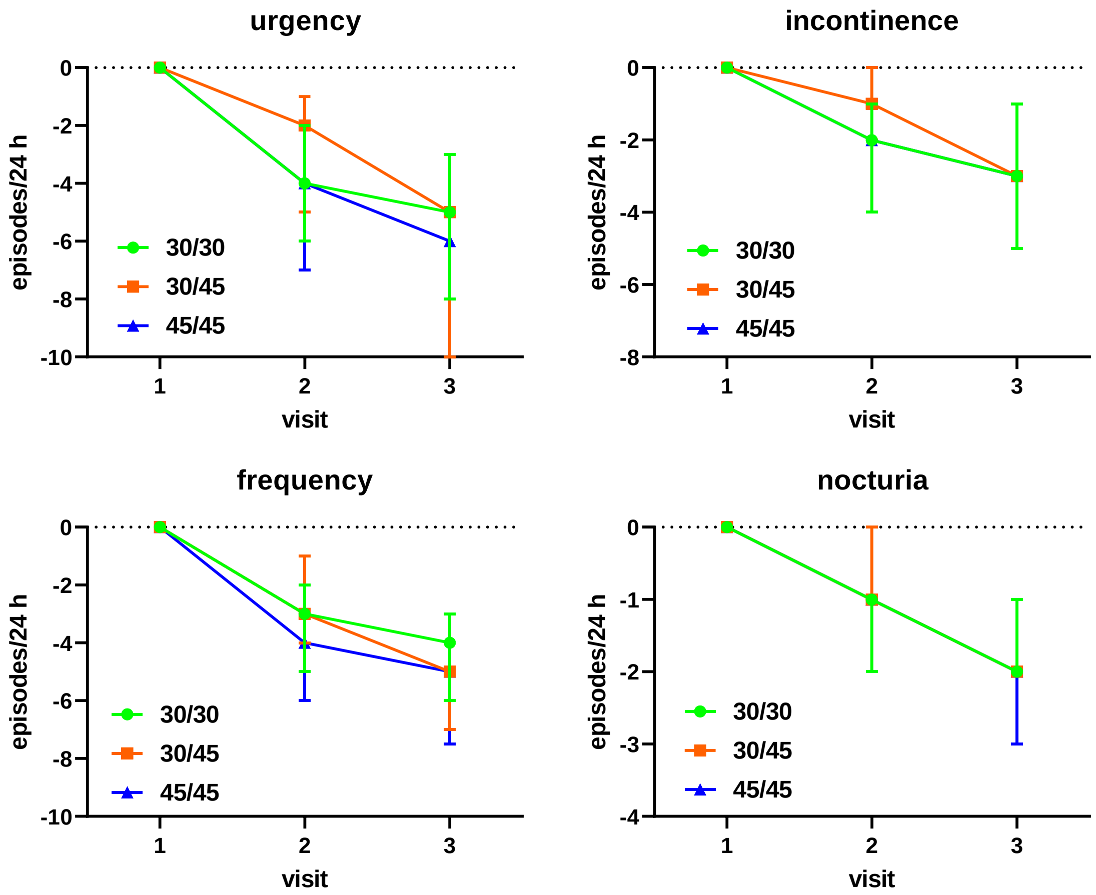

<u>Supplementary Figure 5:</u> Intra-individual change of OAB symptoms in the cohorts of patients starting on 30 mg and staying on that dose (30/30), starting on 30 mg and escalating to 45 mg at visit 2 after about 4 weeks (30/45) and starting and staying on 45 mg until study end after about 12 weeks (45/45) in study II. Patients not exhibiting a given symptom at baseline were excluded from the analysis of that symptom; specifically, 171 subjects reported no incontinence at baseline. Data are shown as means ± SD; medians wit IQR are shown in Supplementary Figure 4.

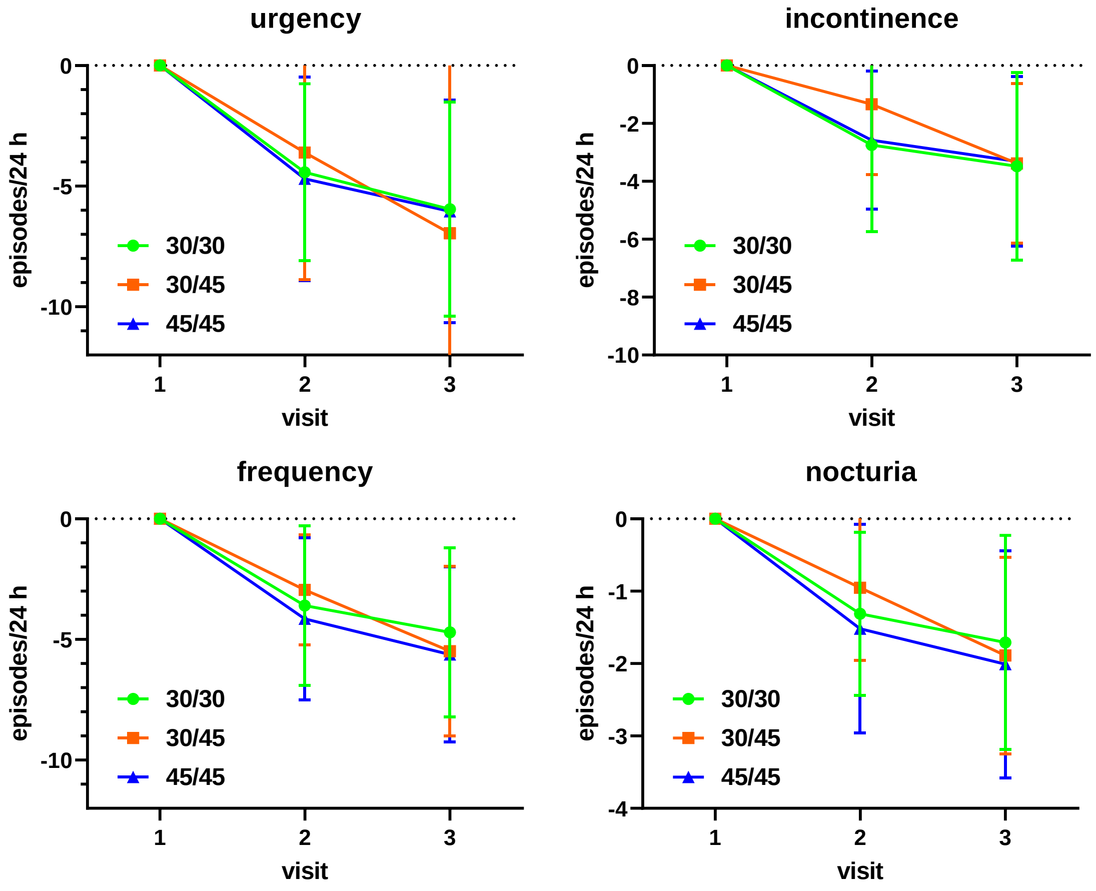

Supplement: Supplementary file 1 [file jcm-10-00311-s001.pdf]
